# Screen Media Usage, Sleep Time and Academic Performance in Adolescents: Clustering a Self-Organizing Maps Analysis

**DOI:** 10.1371/journal.pone.0099478

**Published:** 2014-06-18

**Authors:** Carmen Peiró-Velert, Alexandra Valencia-Peris, Luis M. González, Xavier García-Massó, Pilar Serra-Añó, José Devís-Devís

**Affiliations:** 1 Departament de Didàctica de l’Expressió Musical, Plàstica i Corporal, Universitat de València, Valencia, Spain; 2 Departament d’Educació Física i Esportiva, Universitat de València, Valencia, Spain; 3 Departament de Fisioteràpia, Universitat de València, Valencia, Spain; McGill University, Canada

## Abstract

Screen media usage, sleep time and socio-demographic features are related to adolescents' academic performance, but interrelations are little explored. This paper describes these interrelations and behavioral profiles clustered in low and high academic performance. A nationally representative sample of 3,095 Spanish adolescents, aged 12 to 18, was surveyed on 15 variables linked to the purpose of the study. A Self-Organizing Maps analysis established non-linear interrelationships among these variables and identified behavior patterns in subsequent cluster analyses. Topological interrelationships established from the 15 emerging maps indicated that boys used more passive videogames and computers for playing than girls, who tended to use mobile phones to communicate with others. Adolescents with the highest academic performance were the youngest. They slept more and spent less time using sedentary screen media when compared to those with the lowest performance, and they also showed topological relationships with higher socioeconomic status adolescents. Cluster 1 grouped boys who spent more than 5.5 hours daily using sedentary screen media. Their academic performance was low and they slept an average of 8 hours daily. Cluster 2 gathered girls with an excellent academic performance, who slept nearly 9 hours per day, and devoted less time daily to sedentary screen media. Academic performance was directly related to sleep time and socioeconomic status, but inversely related to overall sedentary screen media usage. Profiles from the two clusters were strongly differentiated by gender, age, sedentary screen media usage, sleep time and academic achievement. Girls with the highest academic results had a medium socioeconomic status in Cluster 2. Findings may contribute to establishing recommendations about the timing and duration of screen media usage in adolescents and appropriate sleep time needed to successfully meet the demands of school academics and to improve interventions targeting to affect behavioral change.

## Introduction

The influence of screen media usage (SMU) on children and adolescents' lifestyles has received growing attention during the last decade due to the potential health consequences. Particularly, screen-related sedentary behaviors have been associated to energy imbalances that increase the risk of overweight and obesity, together with metabolic and cardiovascular diseases among children and adolescents [1]–[3]. Furthermore, it has been suggested that SMU reduces time spent in physical activity [4], although some studies and meta-analyses provide weak or no evidence toward a ‘competing hypothesis’ [5], [6]. Others consider it dependent on the day of the week and the type of media used [7], [8].

Research has also shown that SMU interferes with academic activities, such as studying and reading books, hence having negative consequences on academic performance [9]–[12]. However, other studies have found positive consequences [13] or consequences that differ according to the type of media used [14], [15]. In addition, age, gender and socioeconomic status (SES) are associated to SMU and academic performance, but there is no agreement as to which gender is more at risk [9], [10], [14], [15].

Recent reviews have identified a positive association between SMU and sleep problems or insufficient sleep time [16], [17]. Particularly, delayed bedtime and shorter sleep time appear as the most consistent results associated with excessive media use [16]. Nevertheless, evidence on SMU interference with sleep is contradictory; depending on the type of media that is used [18]. For instance, in a recent study, adolescents who watched TV four or more hours per day were more likely to show sufficient sleep; while those who played video-computer games, or used the computer for something different than school work, two or more hours per day, were more unlikely to show sufficient sleep [19]. Insufficient sleep has also been positively related to poor academic performance [20], [21]. In a recent meta-analysis, sleep duration and school performance were directly associated with having a stronger effect on younger students than on older students. This was also found true to a larger effect in studies that surveyed more boys as opposed to studies that included more girls [22]. Nevertheless, the interrelationship of SMU, particularly regarding sedentary media, and sleep and academic performance is not a common research issue despite the presupposed potential associations between these variables. Among the few, Dworak et al.'s study [23] supports the hypothesis of the negative influence sedentary screen media has on children's sleep, memory and learning. Particularly, TV and computer game exposure affects children's sleep and deteriorates verbal cognitive performance.

The scarcity of studies with a focus on these interrelationships could be due to the limitations of traditional statistical analyses. These analyses establish linear relationships among variables, but in a real world scenario most of the relationships are non-linear. Moreover, traditional statistics reduce their power when the number of variables to be interrelated increases. Therefore, new non-linear statistical analyses are needed to investigate the relationship among different SMU, sleep time, academic performance and socio-demographic characteristics. Self-Organizing Maps (SOM) analysis allows establishing non-linear relationship among a great number of variables. Variables are plotted on maps that improve the visualization and interpretation of these results. Kohonen [24] introduced this method of analysis and since then, more than 5,000 studies have employed this analysis, or a modified version of it, in a wide range of disciplines such as engineering, medicine, biology and economics [25].

The purpose of this cross-sectional study is twofold. Firstly, it describes the relationship among SMU (TV/video/DVD, computer, videogames, mobile), sleep time, academic achievement and socio-demographic characteristics of a youth sample (age, gender and SES) through a SOM analysis. Secondly, it clusters adolescents in order to identify groups of young people with different behavioral profiles from SOM output. Clusters with high and low academic performance are of particular interest in this paper due to the potential impact they receive from SMU, sleep time and socio-demographic variables.

## Materials and Methods

### Ethics statement

Materials and procedures were approved by the individual schools, the school districts and the Ethics Committee of the University of Valencia. Written informed consent forms were obtained from parents or participants 18 years of age.

### Participants

A sample of 3,095 Spanish students, aged 12 to 18, participated in this study as part of a wider project designed to assess physical activity, SMU and other psychosocial variables. It represents 65.7% of response rate from the 4,585 adolescents invited to participate in the study. Three thousand and six adolescents responded to the whole questionnaire, one item had 2.5% of no answer and the rest of items had a less than 1.5% of no answer. In obtaining the sample, a multistage stratified sampling procedure was used after dividing the Spanish territory into six areas, as performed in previous studies [26]. Initially, a randomized sample of ZIP codes from each area was considered as the primary unit of sampling. Public and private schools, the second unit of sampling, were randomly selected among those that offered all educative levels under study and accepted voluntarily to participate. Those schools that refused to participate (3 private and 1 public schools) or did not accomplish the criteria of educative levels were replaced by the first school of the official list of previous ZIP. Adolescents, as ultimate unit of sampling, were selected following proportional quotas by gender and academic course. The field work for data gathering was developed during the Fall of 2010.

### Variables and Instruments

The Adolescent Sedentary Activity Questionnaire (ASAQ) [27] was employed in this study. It is a self-reported instrument to assess the amount of time adolescents spend on daily sedentary behaviors. An intraclass correlation coefficient range of 0.76–0.90 showed an acceptable reliability [27] and a correspondence with accelerometer-determined sedentary time in adolescent girls showed good validity plus test-retest reliability [28]. Only the sedentary SMU variables from ASAQ were used for the purposes of this study and updated variables on mobile use and passive videogames were added. The Family Affluence Scale II [29] was used to measure adolescents' SES. Sleep hours, including a siesta or nap, active videogames usage and age, were questions also included in the study. Responses were recorded as continuous variables and are as follows: a) TV/video/DVD viewing; b) computer for playing; c) computer for communicating; d) computer for doing homework; e) overall computer use (b+c+d); f) passive videogames; g) active videogames; h) mobile for communicating; i) mobile for playing; j) overall sedentary screen media (a+e+f); k) sleep time; l) SES and m) age.

The categorical variables were gender and adolescent academic achievement or performance in the previous academic year, grouped in four categories (1 = more than three failed subjects; 2 = between one and three failed subjects; 3 = no failed subjects and average grades; 4 = no failed subjects and high grades). Categories 1 and 2 correspond to students with poor achievement and are likely to have problems to pass the course. Categories 3 and 4 consist of students with good and excellent achievements that allow them to successfully pass the course.

### Data analysis and procedure

SOM was applied to this study in order to classify adolescents and establish reciprocal links or topological relationships among the considered variables. Therefore, it allowed to group students according to their academic achievement, age, gender, SES, sleep time and SMU. Moreover, SOM also helped to visually analyze the magnitude of every variable. It is an innovative non-linear modeling data analysis, particularly suitable when data are numerous and the equation model that explains the relationships between variables is unknown, as in this study. SOM uses competitive non-supervised neuronal network algorithms [30]. The term competitive means that, when a neuron wins a case, a training of that neuron (and those nearby) occurs, but not of outlying neurons. They are non-supervised because, instead of defining a target variable, all variables are included as input variables.

Matlab R2008a (Mathworks Inc., Inc, USA) was used for data analysis. This software is a high-level language and interactive environment for numerical computation, visualization, and programming. SOM toolbox (2.0 beta version) for Matlab [31] was also employed. This toolbox enabled the performance of different sorts of training to generate the SOM and allowed different visualizations of the results (e.g. u-matrix, component planes). The procedure started with the building of a lattice, composed by nodes or neurons (understood as a mathematical term) with an appropriate size, depending on the input data employed. In order to obtain the final lattice size, the toolbox employed made the calculus in two steps. Firstly, it made an approach of the total size of neurons using the following equation, 5 * sqrt(n). Secondly, the software adjusted the value according to the maps rectangular shape and neurons hexagonal form, resulting in a lattice size of 27×10. Once the lattice size was established, the SOM was initialized by assigning a weight vector to each neuron. Amidst the different types of initializations, we used randomized and linear ones. The first type occurs when the weight vectors are initialized with small random values. The second type happens when the weight vectors are initialized in an orderly fashion along the linear subspace spanned by the two principal eigenvectors of the input data set. Prior to the lattice training, data input was normalized [0…1].

During the training, each neuron competed against the others for every case or participant. The word ‘training’ in this context referred to the set of processes by which the initial weights assigned to the neurons changed, as new cases were added to the analysis. These changes in the weights finished when acquiring a final value representing, with the lowest possible error, all subjects contained in each neuron. The assignation of a case or participant was performed by comparing its value to the value of each neuron from the lattice. The winner neuron was the one that presented the minor Euclidean distance with the participant's values. Once the participant was assigned to the winner neuron, an adaptive process started in which the winner neuron and its neighboring weights were modified and simultaneously organized topographically (i.e. ordering and convergence phases). The neurons nearest the winner neuron exhibit the largest transformations in their values, while the farthest neurons exhibit smaller transformations. This depends on the neighborhood function employed and it is worthy to point out that the adaptation of these weights at the beginning of the learning process was larger than at the end.

1,600 maps were generated in our study as a result of combining two initialization systems (random and linear initialization), two types of training (sequential and batch training), four neighboring functions (Gaussian, cut Gaussian, Epanechicov and Bubble) and 100 initializations. The maps generated in our work showed two types of errors: quantization and topographic. The quantization error expressed how accurate was the value assigned to the neuron regarding the values of each participant who had been included in that neuron. Although adolescents who were included in a neuron had similar values, they were never exactly equal. Consequently, the value assigned to the neuron represented all subjects in a generic way. As the value of the neuron might not be exactly equal to the value of each of the participants, an error emerged.

The topographic error represented the accuracy with which neurons were distributed in the maps. A priori, the process was designed so that the neuron value had similar values to adjacent neurons and different from those in more distant neurons. The process tried to distribute each neuron in the best way. In some cases, the position was performed with greater success and therefore the map showed lower topographical error. Maps can be selected by any of these two criteria (i.e. quantization error or topographical error). In our study, we selected the map that showed the best performance in both parameters, since both errors were important for the interpretation of the results. To ensure that the importance of both errors appeared equally, we decided to multiply both [32].

Eventually, a cluster analysis was performed to establish patterns or profiles among the large groups of participants' responses to different academic performances beyond the small neurons from the SOM [33], [34]. Therefore, a *K*-means method was employed to execute larger cluster divisions, choosing the number of clusters that showed a smaller error. The algorithm k-means is an iterative method to minimize the sum of the square cases belonging to each cluster. Given an initial number of K clusters, this method assigns the cases to each cluster (to the one of Euclidean closer distance). Once assigned the centroid of the cluster, it is calculated as a means of the cases and it assigns again the cases to the centroid. This process is repeated since the problem converges.

Neuronal weights were used for inferential statistical analysis after SOM analysis and cluster identification. Non-parametric analyses were applied since neuronal weight values from the variables under study did not accomplish normality and homoscedasticity assumptions. In particular, Spearman correlations were established between all variables. 95% confidence intervals were presented for correlations in Table 1, and for descriptive data of the different clusters in Table S1.

**Table 1:** Spearman correlations between quantitative variables.

	Age	Academic achievement	Sleep time	TV/vide/DVD	Computer playing	Computer communicating	Computer for doing homework	Overall computer use	Passive videogames	Active videogames	Mobile for communicating	Mobile for playing	Overall sedentary screen media usage
Age	1												
Academic achievement	−0.61	1											
	(−0.69, −0.52)												
Sleep time	−0.97	0.55	1										
	(−0.98, −0.96)	(0.45,0.64)											
TV/video/DVD	−0.16	−0.5	0.15	1									
	(−0.29, −0.03)	(−0.59, −0.39)	(0.02,0.28)										
Computer playing	−0.19	−0.46	0.20	0.61	1								
	(−0.31, −0.06)	(−0.56, −0.35)	(0.07,0.32)	(0.52,0.69)									
Computer communicating	0.4	−0.39	−0.46	0.45	0.03	1							
	(0.28,0.51)	(−0.50, −0.27)	(−0.56, −0.35)	(0.34,0.55)	(−0.10,0.16)								
Computer for doing homework	0.31	−0.37	−0.38	0.47	0.22	0.91	1						
	(0.19,0.42)	(−0.48, −0.25)	(−0.49, −0.26)	(0.36,0.57)	(0.09,0.34)	(0.88,0.93)							
Overall computer use	0.26	−0.48	−0.33	0.61	0.44	0.88	0.91	1					
	(0.13,0.38)	(−0.58, −0.37)	(−0.44, −0.21)	(0.52,0.69)	(0.33,0.54)	(0.85,0.91)	(0.88,0.93)						
Passive videogames	−0.25	−0.35	0.28	0.44	0.92	−0.26	−0.07	0.14	1				
	(−0.37, −0.12)	(−0.46, −0.23)	(0.15,0.40)	(0.33,0.54)	(0.90,0.94)	(−0.38, −0.13)	(−0.20,0.06)	(0.01,0.27)					
Active videogames	−0.73	0.05	0.71	0.52	0.74	−0.28	−0.07	0.06	0.77	1			
	(−0.79, −0.66)	(−0.08,0.18)	(0.64,0.77)	(0.42,0.61)	(0.67,0.79)	(−0.40, −0.15)	(−0.20,0.06)	(−0.07,0.19)	(0.71,0.82)				
Mobile for communicating	0.57	−0.25	−0.61	0.1	−0.49	0.78	0.56	0.47	−0.69	−0.69	1		
	(0.47,0.65)	(−0.37, −0.12)	(−0.69, −0.52)	(−0.03,0.23)	(−0.58, −0.38)	(0.72,0.83)	(0.46,0.64)	(0.36,0.57)	(−0.75, −0.61)	(−0.75, −0.61)			
Mobile for playing	−0.12	−0.5	0.15	0.69	0.81	0.05	0.18	0.34	0.75	0.59	−0.31	1	
	(−0.25,0.01)	(−0.59, −0.39)	(0.02,0.28)	(0.61,0.75)	(0.76,0.85)	(−0.08,0.18)	(0.05,0.31)	(0.22,0.45)	(0.69,0.80)	(0.50,0.67)	(−0.42, −0.19)		
Overall sedentary screen media usage	0.01	−0.55	−0.05	0.89	0.71	0.6	0.68	0.84	0.49	0.45	0.15	0.66	1
	(−0.12,0.14)	(−0.64, −0.45)	(−0.18,0.08)	(0.86,0.91)	(0.64,0.77)	(0.51,0.68)	(0.60,0.75)	(0.80,0.88)	(0.38,0.58)	(0.34,0.55)	(0.02,0.28)	(0.58,0.73)	

Data are correlations coefficients (95%CI).

## Results

**Figure 1:**
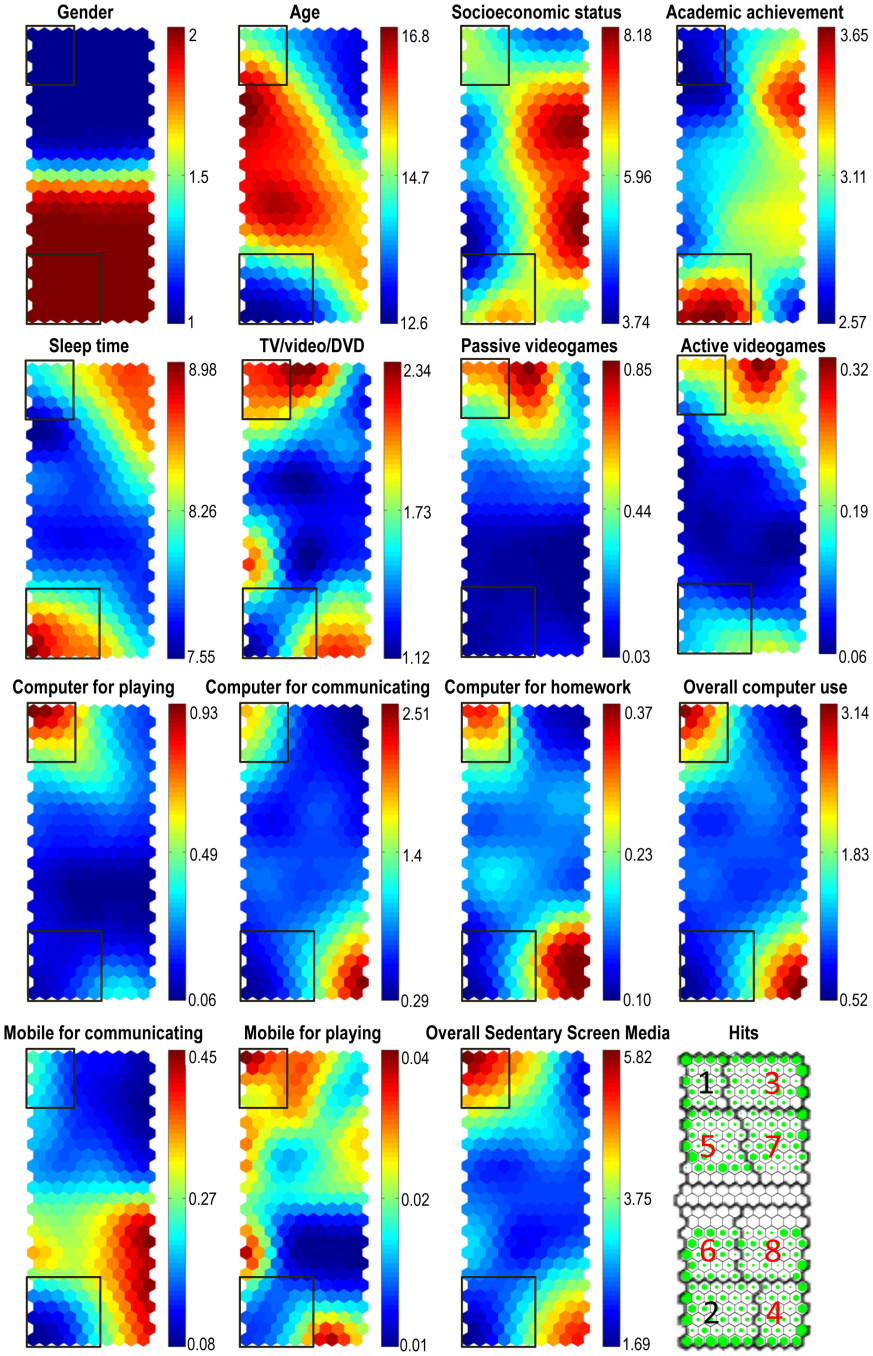
Component planes, hits and target clusters. Hits map can be seen in the right bottom corner. Empty cells show a lack of cases and greener cells indicate a large number of adolescents accumulated in them. Thicker lines separate the different calculated clusters. Numbers 1 and 2 designate target zones or clusters with low and high academic achievements, respectively. Numbers in red (3–8) refer to quantitatively-analyzed clusters with results presented in Table S1 in supplementary data section. The fifteen variables included in the analysis appear from the top to the bottom rows and from the left to the right columns. Black outlined squares in each component plane are drawn to facilitate positions of Clusters 1 and 2. Gender variable is coded as Male =  1 and Female  = 2; Rectangles on the right of each component map indicate the lower (bluish) and higher (reddish) values of each variable. The values in the bottom section of the socioeconomic status and academic achievement variables represent a low socioeconomic status and poor academic achievement. On the contrary, the values at the top of the colored rectangle show a high socioeconomic level and a good academic performance. The rest of the variables are expressed in hours. In order to understand the maps it is important to note that participants included in each neuron (hexagon) are the same in every component plane.

As a result of the SOM analysis, a final group of 15 maps corresponding to the 15 variables of our study emerged (see Figure 1). The visualization of the maps as a whole permitted establishing reciprocal links or topological relationships among them. In particular, gender relationships were easy to visualize because boys were located in the north zone of the maps and girls in the south zone (see the longitudinal side axis of the gender map). According to this, boys used passive videogames and computers for playing to a greater extent than their female counterparts, while girls, more often than boys, used mobile phones to communicate with other people. Other relationships indicated that the more time adolescents used their computer (for any reason), the more time they watched TV/videos/DVDs and played with their mobile phones. Moreover, the oldest adolescents spent less time sleeping and playing with active videogames than their younger counterparts. Finally, the adolescents with the highest academic performance were the youngest and spent more time sleeping and less time using the overall sedentary screen media as compared to those with the lowest academic performance. Moreover, adolescents with higher SES showed direct topological relationship to those having a higher academic performance.

Clustering from the SOM outcomes resulted in nine areas or clusters (see Figure 1, bottom right corner), but only eight were considered because one of them had no subjects in it. Two target clusters, named Cluster 1 and 2, which corresponded to the lower and higher academic performance of both male and female adolescents (see squares indicated in Figure 1), were explored in more detail due to the potential impact caused by SMU, sleep time and socio-demographic features. These clusters gathered 27.2% of the participants from the whole sample and were chosen to identify the particular patterns featured by these groups of adolescents. Descriptive statistics of each cluster are in Table S1.

Cluster 1 was composed of a group of 307 boys, approximately 10% of the sample, with an average age of 15 and a medium SES level. These adolescents spent more than five and a half hours per day using overall sedentary screen media. Their academic performance was low and they slept an average of 8 hours per day. Some participants from this cluster (those grouped in the upper left neuron) used the computer for multi-purpose activities for more than three hours per day (overall computer use), but more hours were used to communicate with people or play (2 hours and a half) than to do their homework (only 15 minutes per day). Besides, it can be observed that participants from this cluster played passive videogames around 30 minutes per day and watched TV/videos/DVDs for more than two hours per day. Regarding the use of the mobile phone, they spent most of their time playing and, occasionally, communicating with others.

The profiles in Cluster 2 showed 537 adolescent girls, 17.3% of the sample, around 13 years old and with a medium SES, whose overall sedentary SMU was much lower than the time consumed by adolescents from the previous cluster. These girls showed an excellent academic performance, a greater sleep time (nearly 9 hours), and less time per day devoted to overall sedentary screen media (two hours and twenty minutes). In fact, they spent very little time watching TV/videos/DVDs, using the computer for playing and using mobile phones to communicate with others.

Regarding non-parametric analysis, Spearman correlations showed a moderate relationship between academic performance and sleep time (r = 0.55), age (r = −0.61) and overall sedentary SMU (r = −0.55). Significant relationships were also found between academic performance and every screen media used (r = −0.25 to −0.50) except active videogames (see Table 1). Correlations between other variables are included in Table 1.

**Figure 2:**
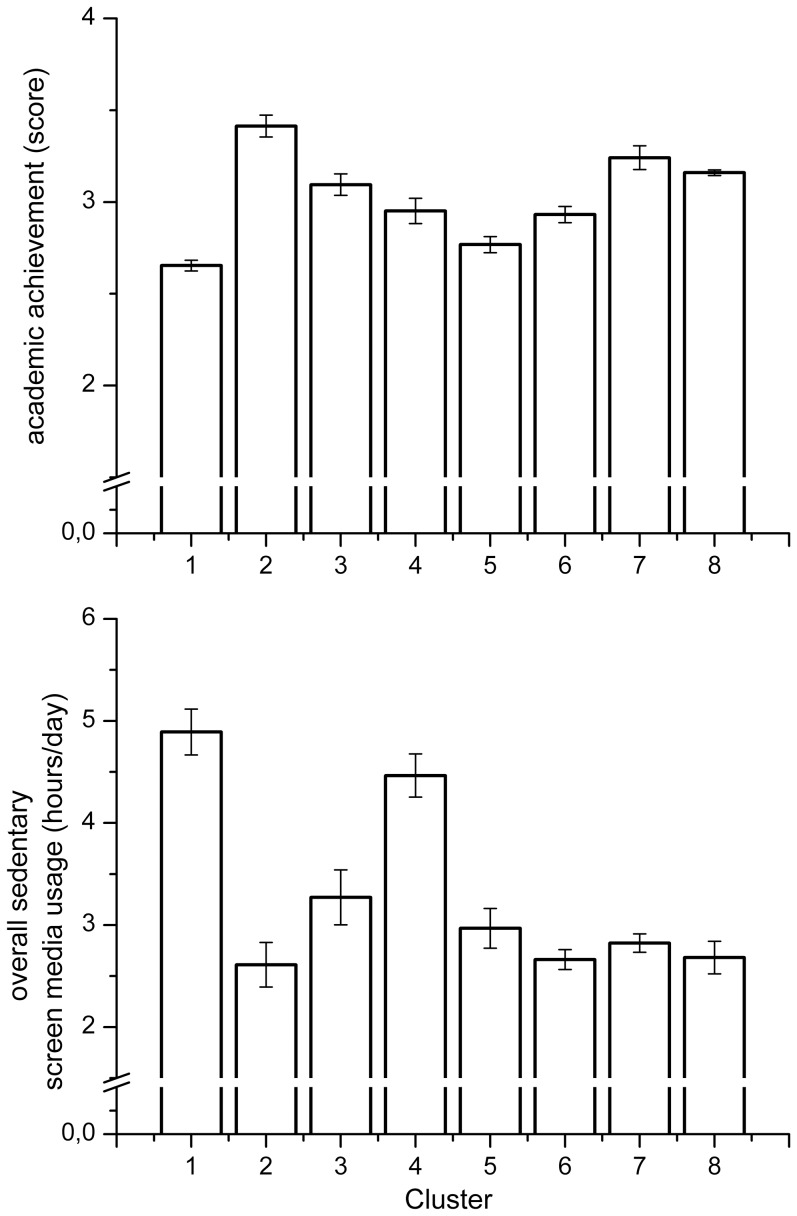
Descriptive statistics for academic performance and overall sedentary SMU variables. Columns represent the mean and error bars represent 95% confidence intervals.

Concerning the analysis of clusters comparison, significant differences were observed among most of them in all dependent variables (see Table S1). According to the main purpose of this study, the interest was focused on academic performance and overall sedentary SMU. As it is observed in Figure 2, academic performance showed significant differences among all clusters except between Cluster 4 and Cluster 6. Overall sedentary SMU revealed higher values of time for Cluster 1 and lower values for Cluster 2. Descriptive data and confidence intervals can be found in the supplementary material (Table S1).

## Discussion

This is one of the few studies that explores the interrelationships among a wide number of SMU, sleep time and academic performance in adolescents [23]. Findings, through visualization of maps from SOM analysis, show a gender differentiation in some SMU. This can be observed, in particular, regarding the purposes of certain media usage. Boys consume more passive videogames, including using computer and mobile phone for playing, while girls use mobile phone more than boys for communicating with others. Age may also affect sleep time and active SMU as the oldest adolescents spend less time sleeping and playing with active videogames than their younger counterparts. Despite cultural and school system differences, the study results on these issues seem to confirm international trends [9], [14], [16], [35].

This study also shows a direct relationship or topological link between overall computer use and TV/video/DVD watching, and mobile phone playing. Furthermore, academic performance is directly related to sleep time and inversely related to overall sedentary SMU among the students who participated in this study.

These results are similar to those carried out in Europe and North America and point out consistently that boys spend more time on the computer and playing videogames than girls [8], [9], [35]–[38]. Girls' higher use of mobile phone for communicating with others is also similar to the North American CHIC study that reports talking on the phone as the primary activity for female adolescents [39]. This is akin to Australian findings where girls devote twice the time to general social activities compared to boys [40]. Due to the novel contribution of this study in distinguishing the different purposes of mobile phone use (for communicating and playing), our result regarding the direct relationship between overall computer use and mobile phone use for playing becomes difficult to link to existing literature. Only a direct relationship between overall computer/videogame use and overall mobile phone use is found in a previous Spanish study [37].

Findings from the present study concerning the direct relationship between overall computer use and TV/video/DVD watching are discordant with those reported in the literature, which indicate that children who use computers may view less TV than nonusers [35] and the presence of a computer in adolescents' bedrooms reduces the amount of TV watched [38]. Nevertheless, the present study indicates the possibility of finding adolescents who spend a lot of time at the computer and watching TV, and both behaviors do not compete with each other. This is likely to happen due to a more interactive character of computer use compared to TV watching. It also occurs in studies that provide weak or no evidence of a competing hypothesis between SMU and physical activity [36].

Contrary to the previous discordant data, in this study the decrease in reported sleep time depending on age is a consistent finding in literature, as several meta-analysis throughout the human lifespan and studies on children and adolescents demonstrate [41], [42]. Active videogame playing, a recent variable in the study of SMU among adolescents, also decreases with age in accordance with a recent Canadian study with students from grades 10 and 11 [43].

Regarding academic performance, overall sedentary SMU is indirectly related to it, either because screen time displaces homework and learning [10], [12], or because it involves intermediate processes that negatively affect academic performance [11]. However, contradictory results emerge concerning computer use. According to a review on the issue and a recent North American study [13], [35], computer use, even for fun and moderate levels of playing games, is positively related to cross-sectional reading and mathematics, and academic achievement or performance. More agreement is found in literature regarding the direct relationship between sleep time and academic achievement in adolescents or between insufficient sleep time and poor academic performance from different countries [11], [20]–[22], [41]. This study also contributes to confirming this international trend, by employing a new methodology, and to informing new profiles unknown so far. In fact, the maps of sleep time and academic performance show a direct topological link and, both of them, an indirect relationship with the age map. A direct relationship was also observed between SES and academic performance, although slightly weaker than in the aforementioned variables. This result confirms the relevance of SES in previous international studies [44], [45] as well as in the recent Spanish results from the Programme for International Student Assessment reported by the OECD [46].

Cluster analysis developed from the SOM outcomes resulted in eight groups of adolescents. For the purpose of this study, two adolescents' profiles (Cluster 1 and 2) were considered as target groups. Cluster 1 features adolescents with the lowest academic performance (i.e. students with and average score of 2 points and difficulties in passing the course) and Cluster 2 represents adolescents with the highest academic performance (i.e. students with an average score of more than 3 points and likely to pass the course with high grades). The two profiles are strongly differentiated by gender, age, sedentary SMU and sleep time. Cluster 1's profile also encompasses the highest use of sedentary screen media, less sleep time compared to girls in Cluster 2, a medium SES and their being the eldest of the sample. The results in Möβle et al. [10] suggest that boys are a group at risk of achieving poor academic performance due to the negative effects extensive use of electronic media has on them, together with being better equipped with screen media devices than girls are and possessing a preference for violent media content. Assuming that other significant variables may affect poor academic achievement, an excessive consumption of these screen media and a lack of proper sleep time may certainly contribute to explaining the effects on school-related achievements. Delineation of this profile should attract a change in strategies aimed to reduce potential school leavers.

Conversely, Cluster 2 achieves the highest academic performance and is comprised of younger girls, with a medium SES, who sleep more time and spend half the amount of time on sedentary SMU than adolescents in Cluster 1. Although they spend an average of two hours and twenty minutes daily on those media and, therefore, exceed the AAP recommendation [47], they are closer to reaching the two hours maximum. This group of girls also uses mobile phones for communicating with others less often than the rest of the girls in the whole sample. Contrary to previous studies [39], [40], this result reveals that speaking on the phone and communication are not their main social activities. Moreover, adolescent girls in this cluster reported sleeping one hour more than the boys in Cluster 1, thus supporting other studies that indicate that girls consistently sleep longer than boys [48].

SES does not emerge as a crucial variable in these two clusters as it does in the overall SOM outcomes. However, it should be mentioned that Cluster 2 has a less homogeneous SES than Cluster 1. A visualization of both SES and academic performance maps indicate that adolescent girls with higher SES obtain the highest academic results.

Some potential weaknesses of this study need to be considered. First, this study used retrospective self-reported data from adolescents that may raise some concern about the reliability and accuracy of the data. However, as far as the ASAQ is concerned, it has been previously proven reliable and valid [27], [49] and has subsequently been used in several studies from different countries [50]–[52]. In a similar vein, the Family Affluence Scale II is an improved version of a previous instrument [29] and it has been used in adolescent samples of international studies [5], [53].

Second, this cross-sectional study presents a description of a situation at a certain moment in adolescents' lives. Therefore, it precludes causal inferences on the associations among their SMU, sleep time, academic performance and some socio-demographic variables and limits the possibility to determine changes over time in all these variables.

Third, the usage of a SOM analysis establishes topological links among variables, while most of the research on these issues commonly employs linear relationships. Therefore, comparisons to other studies should be done with caution. Finally, other interpretations of SOM analysis are also possible. Key zones have been chosen for this paper consistent with the focus of our study (i.e. academic achievement), however other research interests could lead authors to outline different analysis zones and proceed subsequently with the same protocol as followed for this article. For instance, if we set our aim on SES, two zones with the lowest SES values appear just below zone 1 and above zone 2, and on the opposite side, the two highest SES zones. Cluster analysis would assist researchers in defining these zones and establishing new profiles. Indeed, the concision required in academic articles advises against developing a complete analysis and, consequently, these other issues should be approached in future works.

Despite the aforementioned limitations, the study shows some strength for obtaining descriptive information such as its low cost and high feasibility with a large sample of adolescents, as well as the usage of an innovative non-linear analysis of the data. Results from the present study highlight some variations affecting the academic performance of Spanish adolescents due to their socio-demographic characteristics, sleep time and time devoted to SMU. These findings are useful, not only for exploratory purposes, but also for information on behavior patterns that may contribute to establishing recommendations about the timing and duration of the SMU in adolescents and the appropriate sleep time needed to meet school academic demands successfully. In addition, these findings may contribute to improving interventions targeted to affect behavior change and increase academic performance. Particularly, knowledge about Cluster 2 on girls' highest academic performance and about Cluster 1 on boys' poorest academic achievement sheds some light towards understanding and changing adolescent profiles in future intervention studies.

## Supporting Information

Table S1 **Descriptive statistics of the different clusters.**
(XLSX)
